# From Inflammation to Precision Medicine: Mechanistic Insights into Asthma, COPD, and IPF

**DOI:** 10.3390/biomedicines14051055

**Published:** 2026-05-07

**Authors:** Najla Ghrairi, Youssef Zied Elhechmi, Soumaya Ben Saad

**Affiliations:** 1 Faculty of Medicine of Tunis, University of Tunis El Manar, Ariana 2080, Tunisiasoumaya.bensaad@fmt.utm.tn (S.B.S.); 2 Medical Biology Laboratory, Abderrahmen Mami Hospital, Ariana 2080, Tunisia; 3 Intensive Care Department, Habib Thameur Hospital, Tunis 1008, Tunisia; 4 Pulmonology Department PC, Abderrahmen Mami Hospital, Ariana 2080, Tunisia

**Keywords:** non-communicable respiratory diseases, asthma, idiopathic pulmonary fibrosis, chronic obstructive pulmonary disease

## Abstract

Asthma, chronic obstructive pulmonary disease (COPD), and idiopathic pulmonary fibrosis (IPF) are major non-communicable respiratory diseases (NCD-RDs) with high morbidity and mortality. Despite distinct clinical features, they share overlapping mechanisms including oxidative stress, epithelial injury, and immune dysregulation. Asthma is mainly driven by type 2 inflammation, with IL-4, IL-5, and IL-13 inducing eosinophilia, IgE production, mucus hypersecretion, and airway remodeling. Biologics targeting IgE, IL-5, and IL-4Rα have transformed treatment, and agents directed against TSLP and IL-33 further extend the range of targeted interventions. In contrast, COPD involves chronic inflammation with macrophages, neutrophils, and CD8+ T cells, persisting after smoking cessation. Advances include biologics such as dupilumab and benralizumab in eosinophilic COPD, and novel inhaled therapies such as ensifentrine, the first dual PDE3/4 inhibitor delivered via inhalation. IPF, on the other hand, arises from defective epithelial repair and fibroblast activation, causing progressive fibrosis. Approved antifibrotics (nintedanib, pirfenidone) slow lung function decline, while new strategies target TGF-β, CTGF, and fibroblast-directed pathways. Across these diseases, biomarkers and the treatable traits framework are reshaping precision care. Personalized approaches integrating biomarkers, omics, and targeted therapies represent the most promising path for improved outcomes.

## 1. Introduction

Chronic respiratory diseases (CRDs) such as asthma, chronic obstructive pulmonary disease (COPD), and idiopathic pulmonary fibrosis (IPF) are among the most prevalent non-communicable diseases worldwide. Together, they account for a substantial proportion of global morbidity and mortality, with asthma affecting more than 300 million individuals [1], COPD projected to rank as the third leading cause of death [2], and IPF associated with a median survival of only 3–5 years after diagnosis [3]. Beyond their clinical impact, these conditions impose a heavy socioeconomic burden, highlighting the urgent need for improved therapeutic strategies. Although asthma, COPD, and IPF differ in etiology and clinical presentation, they share several key pathological mechanisms. Persistent inflammation, oxidative stress, epithelial injury, and immune dysregulation converge to drive airway or parenchymal remodeling and progressive loss of lung function [4]. Genetic predispositions (such as MUC5B polymorphisms in IPF), environmental exposures (tobacco smoke, air pollution, allergens), and aging processes further contribute to disease heterogeneity [5].

In recent years, precision medicine has reshaped the therapeutic landscape of chronic respiratory diseases. In asthma, biologics targeting IgE, IL-5, and IL-4/IL-13 pathways have revolutionized the management of severe, type-2 high disease. In COPD, blood eosinophil counts are now recognized as biomarkers to guide inhaled corticosteroid use, and recent advances include targeted biologics such as dupilumab for eosinophilic phenotypes. In IPF, antifibrotic agents pirfenidone and nintedanib remain the cornerstone of therapy, effectively slowing lung function decline [3,6]. However, novel insights into pro-fibrotic signaling, including TGF-β and CTGF pathways, as well as emerging approaches such as LPA1 antagonists and integrin inhibitors, highlight ongoing innovation and unmet needs [6]. This review aims to provide an updated synthesis of shared inflammatory pathways, emerging biomarkers, and therapeutic innovations in asthma, COPD, and IPF. We further discuss the Treatable Traits framework as a unifying model for precision respiratory medicine, bridging mechanistic insights with clinical translation to improve outcomes in these debilitating diseases.

## 2. Shared and Distinct Inflammatory Pathways in Asthma, COPD, and Idiopathic Pulmonary Fibrosis

### 2.1. Asthma: Type 2 Inflammation and Immune Heterogeneity

Asthma is a heterogeneous chronic inflammatory airway disease marked by reversible airflow obstruction, airway hyperresponsiveness, and varied clinical endotypes. The most prominent pattern is type 2 inflammation, orchestrated by Th2 cells and group 2 innate lymphoid cells (ILC2s), which secrete IL-4, IL-5, and IL-13. Collectively these cytokines drive eosinophilic recruitment, IgE class switching, mucus hypersecretion, and subepithelial fibrosis, all contributing significantly to airway remodeling and persistent symptoms [7,8,9].

**Figure 1:**
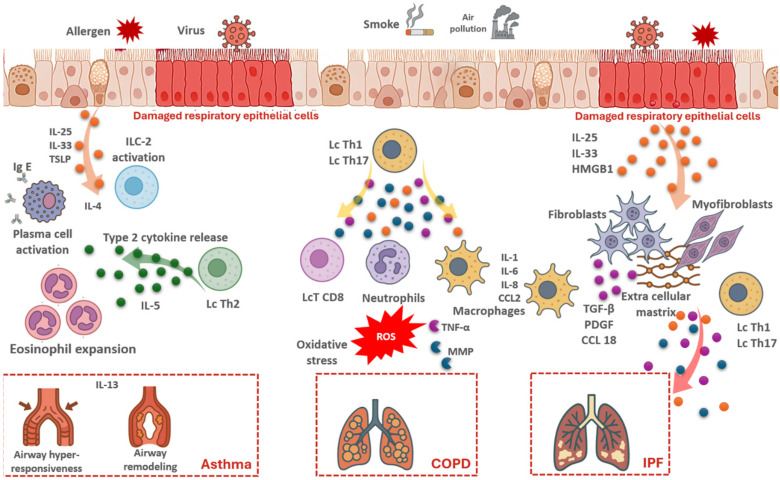
Distinct inflammatory mechanisms in asthma, COPD, and IPF. Schematic representation of disease-specific pathways: type 2 inflammation and airway remodeling in asthma; neutrophilic inflammation, oxidative stress, and emphysema in COPD; epithelial injury, fibroblast activation, and fibrosis in IPF. Abbreviations: IL, interleukin; TSLP, thymic stromal lymphopoietin; HMGB1, high-mobility group box 1; ILC-2, group 2 innate lymphoid cells; Th, T helper cells; CD8+ T cells, cytotoxic T lymphocytes; ROS, reactive oxygen species; TNF-α, tumor necrosis factor alpha; MMP, matrix metalloproteinase; TGF-β, transforming growth factor beta; PDGF, platelet-derived growth factor; CCL2/CCL18, C-C motif chemokines; ECM, extracellular matrix; IgE, immunoglobulin E; DC, dendritic cells.

IL-5 promotes eosinophil survival, IL-13 induces goblet cell hyperplasia, and IL-4 is essential for IgE class switching. Upstream alarmins (IL-33, TSLP, IL-25) enhance these responses by activating ILC2s and dendritic cells [10,11,12]. Epithelial barrier dysfunction amplifies this process: environmental allergens and pollutants trigger epithelial damage, alarmin release, and polarization of naïve T cells toward a Th2 profile, perpetuating chronic inflammation and structural remodeling [13]. In genetically predisposed individuals, dysregulated epithelial–immune interactions sustain this cycle. These mechanisms are summarized schematically (see Figure 1). Beyond T2-high disease, non-type 2 phenotypes (neutrophilic or paucigranulocytic) involve Th1/Th17 pathways, are often corticosteroid-resistant, and contribute to severe forms of asthma. Recognition of these endotypes has enabled the development of biologics such as omalizumab (anti-IgE), mepolizumab/benralizumab (anti-IL-5), and dupilumab (anti-IL-4Rα), which provide tailored control based on immune profiling [14,15].

### 2.2. Chronic Obstructive Pulmonary Disease (COPD): Persistent and Heterogeneous Inflammation

In contrast to asthma, COPD is dominated by chronic macrophage, neutrophil, and CD8+ T-cell inflammation, largely triggered by smoking or biomass exposure, and persisting even after exposure cessation. Alveolar macrophages—5–10 times more abundant than in healthy lungs—produce TNF-α, IL-1β, IL-6, IL-8, and matrix metalloproteinases (MMP-9, MMP-12), contributing to extracellular matrix degradation and emphysema [16].

Neutrophils, recruited by chemokines such as CXCL8/IL-8, release elastase, reactive oxygen species, and NETs, thereby aggravating oxidative stress and epithelial injury [17]. CD8+ T cells accelerate emphysema progression, while B-cell follicles indicate an autoimmune component [18,19]. The cellular and molecular mechanisms underlying COPD are illustrated in Figure 1.

Notably, 30–40% of COPD patients exhibit eosinophilic inflammation, overlapping with T2 pathways classically described in asthma. These patients often respond better to corticosteroids and may benefit from biologics targeting IL-5 or IL-4Rα [20]. This highlights both the shared pathways (IL-4, IL-5, IL-13 involvement) and the distinct dominance of neutrophil/macrophage inflammation in COPD, supporting stratified therapeutic approaches.

### 2.3. Idiopathic Pulmonary Fibrosis (IPF): Dysregulated Inflammation and Aberrant Wound Repair

Unlike asthma and COPD, IPF is not primarily inflammation-driven but results from defective epithelial repair and uncontrolled fibroblast activation. Recurrent epithelial micro-injuries linked to environmental factors, aging, oxidative stress, or MUC5B polymorphisms induce the release of alarmins such as IL-33, TSLP, and HMGB1 [21,22]. These signals stimulate fibroblasts and myofibroblasts to produce collagen I and fibronectin, forming fibrotic foci.

Although inflammation is secondary, alternatively activated macrophages and Th2/Th17 subsets contribute by secreting pro-fibrotic mediators (e.g., TGF-β1, PDGF, CCL18), which amplify fibroblast proliferation and extracellular matrix deposition [21]. A schematic representation of these processes is shown in Figure 1.

Persistent low-grade inflammation may partly explain heterogeneous disease courses and occasional corticosteroid responsiveness. Interactions with aging-related mechanisms (telomere shortening, senescence) further accelerate progression [23]. Approved antifibrotics (pirfenidone, nintedanib) slow disease progression, while new therapies aim to modulate fibroblast–immune crosstalk [24,25].

**Figure 2:**
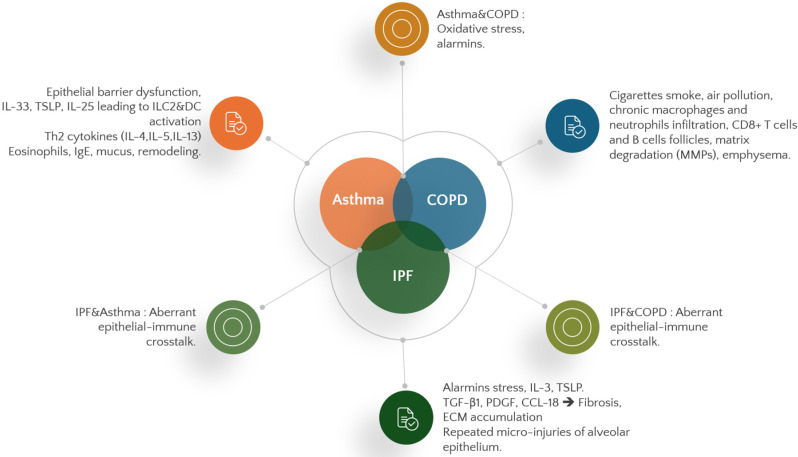
Shared and distinct inflammatory mechanisms in asthma, COPD, and idiopathic pulmonary fibrosis (IPF). Venn diagram showing overlapping mechanisms (oxidative stress, alarmins, profibrotic mediators) and distinct disease-specific drivers.

These disease-specific mechanisms are summarized schematically (see Figure 1) and comparatively (see Figure 2).

## 3. Inflammatory Biomarkers: Endotyping and Precision Treatment

Biomarkers are increasingly central to distinguishing endotypes and guiding targeted therapies.

- Asthma: T2-high phenotypes are defined by blood and sputum eosinophils, FeNO, serum periostin, and total IgE—all predictive of corticosteroid and biologic responses [26]. Consensus guidelines recommend combined FeNO and eosinophil assessment for improved predictive accuracy [27].
- COPD: Blood eosinophils (≥300 cells/µL) predict corticosteroid response and lower mortality in exacerbators [2,28]. Other candidates (CRP, fibrinogen, soluble TNF receptors, neutrophil elastase, MPO, MMPs, SP-D) are under study but remain limited by specificity [29].
- IPF: KL-6, SP-D, and MMP-7 are validated prognostic markers, associated with disease progression and mortality [30,31,32]. Combining KL-6 and MMP-7 improves prediction accuracy. Emerging markers such as CA19-9, periostin, and CCL18 show potential for treatment monitoring, particularly during antifibrotic therapy [32,33].

These comparative features are summarized in Table 1.

## 4. Targeted Therapies: Precision Medicine Across Asthma, COPD, and IPF

**Figure 3:**
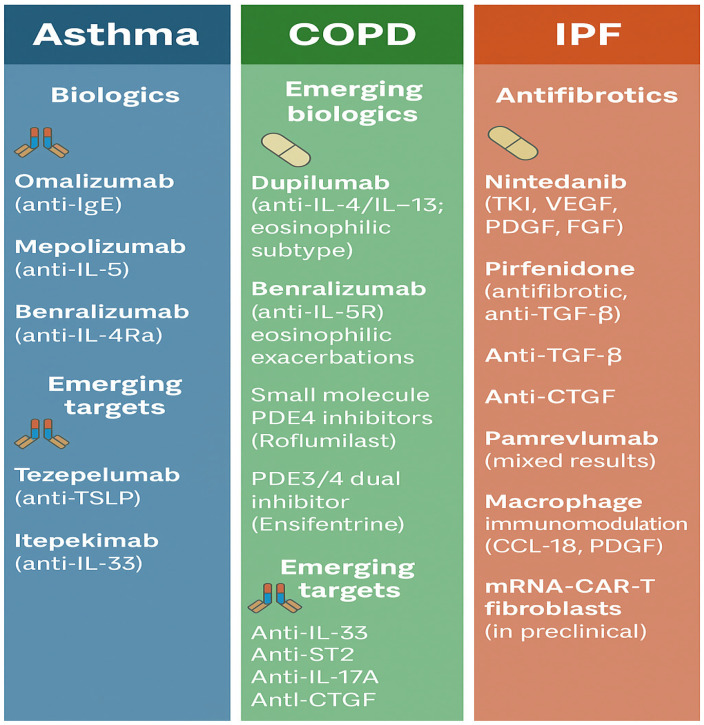
Targeted and emerging therapies across asthma, COPD, and idiopathic pulmonary fibrosis (IPF). Overview of approved biologics, antifibrotics, and novel molecular targets under clinical evaluation.

Advances in targeted therapies now allow clinicians to tailor treatment to underlying immunological endotypes of chronic respiratory diseases. An overview of therapeutic targets across asthma, COPD, and IPF is illustrated in Figure 3.

### 4.1. Targeted Therapies in Asthma

Advances in immunopathology and molecular profiling have revolutionized the management of severe asthma, moving from a uniform approach toward precision medicine. Severe asthma is now recognized as a heterogeneous disease with distinct phenotypes and endotypes, notably T2-high (eosinophilic, allergic) and T2-low (neutrophilic, pauci-granulocytic), each driven by unique immunologic pathways [34,35,36].

Biologic therapies have emerged as central components of the personalized management of T2-high asthma. These agents target key cytokines and pathways involved in eosinophilic inflammation, IgE-mediated responses, and epithelial alarmins.

- ** Anti-IgE therapy (omalizumab): Established as the gold standard for allergic asthma with elevated serum IgE, omalizumab reduces exacerbations, improves quality of life, and decreases inhaled corticosteroid (ICS) requirements in sensitized patients, with efficacy confirmed in both randomized trials and real-world studies [37].
- ** Anti–IL-5/IL-5R therapies (mepolizumab, reslizumab, benralizumab): Initially assessed in patients with baseline blood eosinophilia ≥150–300 cells/µL, these agents demonstrated consistent reductions in exacerbation rates, improved lung function, and corticosteroid sparing. Importantly, subsequent analyses and pivotal trials—including DREAM and MENSA for mepolizumab [38,39,40,41]. SIROCCO and CALIMA for benralizumab [40], and the BREATH program for reslizumab [41]—have shown efficacy across broader eosinophil thresholds, highlighting their robustness beyond initial cut-offs.
- ** Anti–IL-4Rα therapy (dupilumab): By blocking IL-4 and IL-13 signaling, dupilumab has proven effective in eosinophilic and corticosteroid-dependent asthma, improving lung function and reducing exacerbations independently of allergic status [42,43].
- ** Anti-alarmin therapies: These represent the newest therapeutic frontier, though their positioning differs. Tezepelumab (anti-TSLP) is the first biologic to demonstrate efficacy across a wide spectrum of asthma phenotypes, including low-eosinophil subgroups. Data from PATHWAY and NAVIGATOR trials confirmed reductions in exacerbations and broad applicability [44,45,46]. In contrast, itepekimab (anti-IL-33) remains in early development; phase II results are promising, but its role in clinical practice is not yet established [47].

Beyond RCTs, real-world evidence further supports the safety and effectiveness of biologics. Large observational cohorts confirm benefits of omalizumab [48], mepolizumab [49], benralizumab [50], and dupilumab [51], in more heterogeneous populations than those studied in trials. These findings underscore the need to integrate both trial and real-world data to refine patient eligibility and optimize therapy selection.

Despite these advances, challenges persist in non-T2 asthma, where reliable biomarkers remain elusive. Future progress will depend on integrating multi-omics, sputum profiling, and clinical phenotyping to develop the next generation of precision strategies [7,52,53].

A comparative summary of the main biologic therapies in severe asthma, including their mechanisms of action, key eligibility biomarkers, pivotal clinical trials, and current status, is presented in Table 2.

### 4.2. Targeted Therapies in COPD

Recent advances in immunopathology have reshaped our understanding of COPD as a heterogeneous disease with multiple inflammatory profiles. In particular, eosinophilic inflammation has been recognized as a treatable trait in a substantial subset of patients. According to the GOLD 2023 strategy, blood eosinophil counts are now recommended as biomarkers to guide inhaled corticosteroid (ICS) use in patients with frequent exacerbations, with thresholds ≥ 300 cells/µL strongly predicting benefit, and ≥100 cells/µL providing intermediate guidance [2]. This biomarker-driven approach has refined therapeutic strategies and set the stage for precision medicine in COPD.

#### 4.2.1. Biologics Targeting Type 2 Pathways

The concept of using biologics—previously limited to asthma—has expanded into COPD

- ** Dupilumab (anti–IL-4Rα): The landmark NOTUS trial demonstrated a 30–34% reduction in exacerbations, along with improvements in lung function and quality of life, in eosinophilic COPD patients. These results led to FDA approval in 2023, establishing dupilumab as the first biologic indicated for COPD [54].
- ** Benralizumab (anti–IL-5Rα): The ABRA trial, a phase II study, showed reduced treatment failure and improved symptoms following a single 100 mg subcutaneous dose during acute eosinophilic exacerbations [55]. However, larger phase III trials (GALATHEA, TERRANOVA) did not meet their primary endpoints, although post hoc analyses suggested benefit in highly selected eosinophilic subgroups [56].
- ** Mepolizumab (anti–IL-5): The METREX trial demonstrated modest reductions in exacerbations in eosinophilic COPD, whereas METREO failed to confirm consistent benefit [57].

These mixed results highlight the complexity of extrapolating asthma biomarkers to COPD and reinforce the need for refined stratification.

Taken together, these findings illustrate that anti–IL-5/IL-5R therapies may offer benefit only in carefully selected eosinophilic phenotypes, whereas dupilumab has shown broader efficacy, confirming IL-4/13 pathways as more robust therapeutic targets in COPD.

#### 4.2.2. Beyond Type 2 Inflammation

While type 2 pathways are relevant in 30–40% of patients, non-T2 inflammation dominates in many COPD phenotypes. Neutrophilic inflammation, oxidative stress, and activation of inflammasomes such as NLRP3 drive persistent airway injury, emphysema, and corticosteroid resistance [58]. These mechanisms, often ICS-insensitive, may explain therapeutic heterogeneity and support the development of new anti-inflammatory targets.

- ** Ensifentrine (dual PDE3/4 inhibitor): Delivered via nebulizer, ensifentrine achieved ~40% reduction in exacerbations and improved lung function in the ENHANCE-1 and ENHANCE-2 trials, marking the most significant inhaled therapy innovation in two decades [59].
- ** Emerging upstream targets: Novel strategies include blocking IL-33 (itepekimab), ST2 (astegolimab), and IL-17A. Although still in early phases, these agents hold promise for addressing steroid-unresponsive and frequent-exacerbator phenotypes [52].

#### 4.2.3. Comparative Perspective

Overall, biologic therapies in COPD have shown more variable efficacy than in asthma, reflecting greater disease heterogeneity. Dupilumab stands out with consistent benefit, while IL-5/IL-5R–directed therapies yield mixed outcomes, and novel anti-inflammatory approaches remain investigational

An overview of biologic and targeted therapies evaluated in COPD, detailing their mechanisms, pivotal clinical trials, main outcomes, and current regulatory status, is provided in Table 3.

### 4.3. Targeted Therapies in IPF

Idiopathic pulmonary fibrosis (IPF) remains a devastating disease, with antifibrotic agents nintedanib and pirfenidone constituting the current standard of care. Both drugs slow the rate of forced vital capacity (FVC) decline but do not halt or reverse established fibrosis. Mechanistically, nintedanib is a tyrosine kinase inhibitor targeting VEGFR, FGFR, and PDGFR pathways, thereby attenuating fibroblast proliferation and extracellular matrix (ECM) deposition. Pirfenidone exerts anti-fibrotic and anti-inflammatory effects, in part through suppression of TGF-β signaling, reduced fibroblast activation, and decreased pro-inflammatory cytokine production [60,61].

Despite these advances, therapeutic progress is challenging. For example, pamrevlumab, a monoclonal antibody against connective tissue growth factor (CTGF), failed to meet its primary endpoint in the phase III ZEPHYRUS-1 trial and the companion ZEPHYRUS-2 was discontinued [62]. Similarly, ziritaxestat, an autotaxin inhibitor expected to reduce lysophosphatidic acid-mediated fibroblast activation, was discontinued after the ISABELA 1/2 trials showed lack of efficacy and safety concerns [63]. These failures highlight the complexity and redundancy of fibrogenic signaling, where blocking a single pathway may not suffice to alter disease trajectory.

In contrast, new molecular strategies are under active evaluation. Admilparant (BMS-986278), a first-in-class lysophosphatidic acid receptor-1 (LPA1) antagonist, has shown in clinical studies a favorable safety profile and significant biomarker modulation, with early evidence of reducing disease progression in idiopathic pulmonary fibrosis (IPF) and progressive pulmonary fibrosis (PPF). Phase III trials are currently underway to confirm these findings [64]. Beyond single targets, other emerging approaches include phosphodiesterase-4 inhibitors, additional tyrosine kinase inhibitors, integrin antagonists (αvβ6, αvβ1), and inhaled prostacyclin analogues such as treprostinil, currently under investigation in the TETON phase III program [65].

Beyond conventional targets, immunomodulatory strategies represent an emerging frontier. Preclinical data on FAP-targeted CAR-T therapy, generated in vivo using lipid nanoparticle–messenger RNA (LNP-mRNA), demonstrated potent anti-fibrotic effects by selectively depleting activated fibroblasts, restoring ECM balance, and promoting alveolar regeneration [66]. In parallel, rentosertib (ISM001-055), a first-in-class TNIK inhibitor discovered using AI-based drug design, has advanced to phase IIa clinical testing in IPF. Preliminary findings indicate good tolerability with early signals of slowed FVC decline compared with placebo [67]. Together, these therapeutic strategies reflect a shift towards precision medicine in IPF, moving beyond traditional antifibrotics to multifaceted approaches targeting epithelial injury, immune dysregulation, and ECM remodeling. The contrast between successful antifibrotics and failed targeted agents underscores the complexity of fibrotic pathways and the need for combination or sequential strategies to effectively modify disease outcomes.

Table 4 summarizes the emerging and targeted therapies in idiopathic pulmonary fibrosis (IPF), highlighting their mechanisms of action, key clinical trials, main findings, and current stage of development.

## 5. Treatable Traits: A Precision Care Framework for Asthma, COPD, and IPF

The Treatable Traits (TTs) paradigm has emerged as a cornerstone of precision medicine in chronic respiratory diseases, aiming to identify disease-relevant, measurable, and modifiable traits across pulmonary, extrapulmonary, and behavioral domains. Unlike guideline-driven care, TT-based strategies emphasize multidimensional assessment, encompassing clinical history, physiology, imaging, biomarkers, comorbidities, and lifestyle factors, followed by targeted interventions delivered through multidisciplinary teams. This approach has been associated with better health-related quality of life and improved symptom control compared with guideline-only care [68,69].

### 5.1. Pulmonary Traits and Biomarker-Guided Therapy

In asthma, the best-defined trait remains type 2–high inflammation, typically identified through blood eosinophils, fractional exhaled nitric oxide (FeNO), and, where available, sputum eosinophils. These biomarkers not only guide the prescription of ICS and biologics but also predict future exacerbation risk. Importantly, their interpretation requires clinical context, since oral corticosteroid use, recent exacerbations, or comorbidities may distort values. GINA 2024 therefore emphasizes biomarker gradients rather than fixed cut-offs, to avoid oversimplification [1].

In COPD, the GOLD 2023 framework integrates blood eosinophil counts as key indicators of ICS responsiveness. Rather than defining rigid cut-offs, eosinophil counts are seen as ranges of benefit: ≥300 cells/µL predicts the highest benefit, while 100–300 cells/µL indicates intermediate responsiveness. This nuance helps clinicians balance ICS benefits in reducing exacerbations against risks such as pneumonia [2].

In IPF and fibrosing ILDs, the TT approach is still emerging. Pulmonary traits currently focus on functional decline (FVC loss, impaired diffusion), radiographic progression, and small airway involvement. Although no single biomarker directly guides treatment, trait-based stratification is being explored to optimize the timing of antifibrotic therapy and to trigger supportive measures such as oxygen supplementation, pulmonary rehabilitation, or transplantation referral [2].

### 5.2. Extrapulmonary Traits: Impact Across Diseases

**Figure 4:**
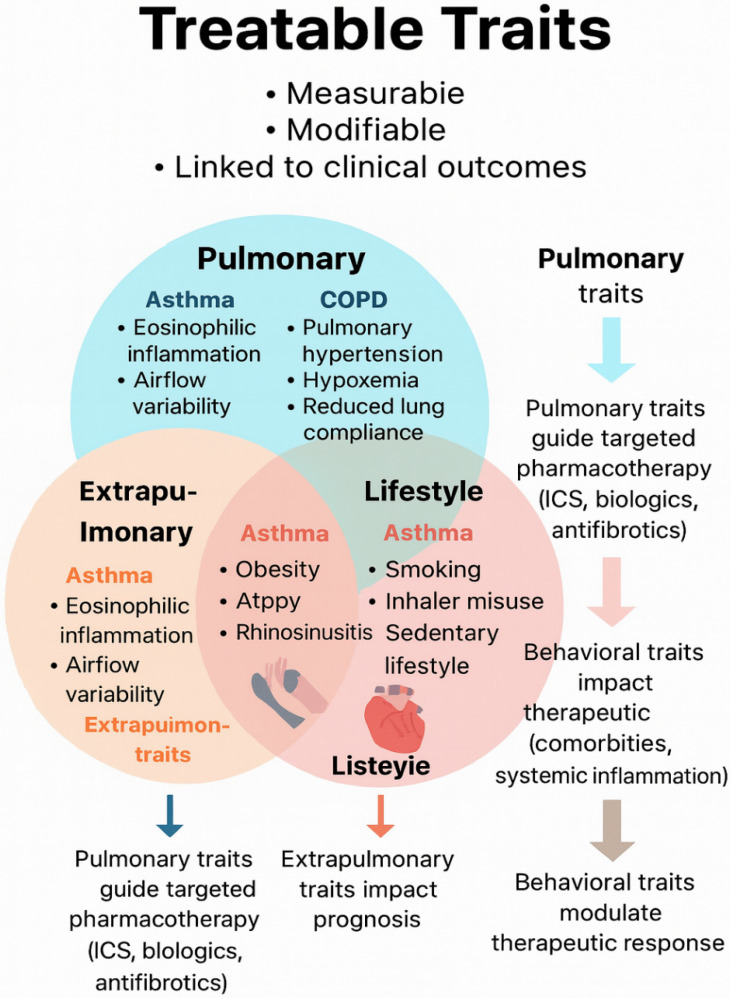
Treatable traits framework in chronic respiratory diseases. Three domains of treatable traits (pulmonary, extrapulmonary, lifestyle) and their impact on therapy and outcomes.

Beyond lung-centered traits, extrapulmonary traits profoundly influence outcomes. In asthma and COPD, obesity, anxiety/depression, sleep-disordered breathing, deconditioning, and systemic inflammation are common contributors to poor control, exacerbations, and higher healthcare utilization. For example, structured weight-loss and exercise programs in obese asthma have shown to improve symptom control and reduce inflammation [70,71]. In COPD–OSA overlap, CPAP treatment improves symptoms and reduces exacerbations, supporting routine screening and treatment of this trait [72]. In ILDs, extrapulmonary traits such as pulmonary hypertension, GERD, frailty, and malnutrition are increasingly integrated into TT frameworks, as they are modifiable and directly affect survival and quality of life [73] (see Figure 4).

### 5.3. Behavioral and Lifestyle Traits

Behavioral traits are highly prevalent and often overlooked. Incorrect inhaler technique, poor adherence, persistent smoking, and physical inactivity significantly reduce treatment effectiveness. Large evaluations reveal frequent inhaler misuse in both COPD and asthma, particularly during hospitalizations. Consequently, TT programs emphasize inhaler education, adherence monitoring, smoking cessation, and exercise training as core components of care [74].

### 5.4. Implementation and Future Directions

The implementation of TT care relies on a structured workflow: standardized intake (symptom scores, exacerbation history, biomarkers, spirometry, comorbidity screening), mapping of traits, and delivery of trait-specific interventions. Recent consensus statements propose algorithms applicable to both primary and specialist care, highlighting the role of digital tools and multidisciplinary clinics to ensure long-term monitoring [68,69].

Looking ahead, the consolidation of TT frameworks requires:

- Standardization of trait definitions and cut-offs across diseases;
- Validation of composite indices integrating clinical, physiological, and biomarker data;
- Integration of omics-enabled phenotyping and AI-supported decision tools;
- Development of prospective TT-guided trials across asthma, COPD, and ILD populations.

Together, these steps will help operationalize the Treatable Traits model into routine practice, transforming care by systematically linking specific traits to targeted interventions and, ultimately, to improved outcomes across chronic respiratory diseases [73].

## 6. Challenges and Future Perspectives

Despite remarkable progress in the understanding and treatment of asthma, COPD, and IPF, the translation of precision medicine into routine clinical practice continues to face major barriers. These challenges arise at multiple levels—biological, methodological, and organizational—and must be addressed to ensure that novel insights are transformed into tangible patient benefits.

### 6.1. Disease Heterogeneity

One of the most persistent obstacles is the marked heterogeneity of these diseases. Traditional diagnostic labels such as “asthma,” “COPD,” or “IPF” often obscure profound within-disease variability. For example, COPD encompasses eosinophilic, neutrophilic, and mixed inflammatory profiles, each with different therapeutic responsiveness, while IPF exhibits diverse trajectories influenced by genetic predisposition, immune dysregulation, and environmental triggers. This complexity underscores the need for frameworks such as Treatable Traits or endotype-driven classifications, which move beyond one-size-fits-all paradigms and enable patient stratification based on mechanistic signatures [73].

### 6.2. Biomarker Validation

Another major challenge is the validation of biomarkers. Although several promising candidates—such as KL-6, SP-D, CXCL4, and anti-MDA5 autoantibodies—have shown diagnostic or prognostic potential, most remain at the investigational stage. Variability between assays, lack of standardized thresholds, and insufficient longitudinal validation hinder their adoption in clinical practice. Until robust harmonization is achieved, the use of biomarkers in routine care will remain limited and uneven across settings [73].

### 6.3. Bridging Translational Gaps

Preclinical models such as precision-cut lung slices (PCLS) and lung organoids provide unique opportunities to study disease mechanisms and test candidate therapies in human-like systems. However, very few molecules identified through these platforms have advanced to successful clinical trials. This translational gap reflects both the complexity of disease biology and the limitations of conventional trial design. Innovative approaches—including adaptive trial methodologies and biomarker-enriched enrollment—are urgently needed to accelerate the transition from preclinical discovery to clinical application [61].

### 6.4. Integration of AI and Multi-Omics

The rise of multi-omics technologies (genomics, transcriptomics, proteomics, metabolomics) combined with artificial intelligence has created unprecedented opportunities for patient stratification and predictive modeling. Yet, practical challenges remain substantial. Data heterogeneity, reproducibility concerns, and the “black-box” nature of many algorithms limit clinical acceptance. Moreover, ethical questions surrounding privacy and bias must be addressed. Translating complex multi-omics signatures into clinically actionable decisions is therefore a central hurdle in the next stage of precision medicine [73].

### 6.5. Regulatory and Logistical Barriers

Even when effective targets are identified, regulatory and logistical challenges slow implementation. Novel upstream therapies directed at mediators such as IL-33, ST2, and TGF-β require long-term safety data, economic justification, and approval processes that are often protracted. In parallel, the implementation of precision medicine requires access to biomarker testing, multidisciplinary infrastructure, and robust patient registries—resources that remain unequally distributed worldwide [73].

### 6.6. Future Perspectives

Moving forward, the construction of a precision medicine ecosystem is paramount. Such a system would integrate TT frameworks, validated biomarkers, omics-enabled phenotyping, and AI-based decision support into routine care [61].

Patient-centered models must be emphasized, as they have already demonstrated improved outcomes in asthma and COPD, and are beginning to shape care in IPF and fibrosing ILDs [73].

Innovative imaging modalities (e.g., αvβ6-integrin PET), quantitative CT metrics, and epigenetic biomarkers are poised to further refine diagnosis and prognosis [61].

In conclusion, the translation of precision medicine into daily respiratory care requires overcoming scientific, regulatory, and infrastructural barriers. By consolidating biomarker validation, integrating digital and omics technologies, and fostering patient-centered frameworks, the field can move from fragmented advances toward a sustainable and equitable model of precision care across asthma, COPD, and IPF.

## 7. Conclusions

Asthma, COPD, and IPF are distinct yet interconnected through shared mechanisms of chronic inflammation, immune dysregulation, and tissue remodeling. This comparative synthesis emphasizes the emerging concept that respiratory diseases exist on a mechanistic continuum rather than within rigid diagnostic boundaries [4]. The evolution toward precision medicine, guided by biomarkers, molecular signatures, and treatable traits, marks a paradigm shift in respiratory care [75]. The success of biologics in severe asthma and eosinophilic COPD, together with antifibrotics in IPF, exemplifies the clinical translation of pathophysiological insights into targeted therapies. Moreover, novel interventions such as integrin inhibitors, senescence-targeting compounds, and immunomodulatory agents hold promise for further breakthroughs across these diseases [35,76]. The originality of this review lies in its integrative perspective, linking shared biological pathways to disease-specific therapeutic strategies. Clinically, it underscores the need for a multidimensional, patient-centered framework that combines clinical phenotyping, multi-omics profiling, and artificial intelligence-based tools to enable real-time personalization of care. Ultimately, such an approach may redefine prevention, diagnosis, and treatment across the spectrum of chronic respiratory diseases, paving the way toward truly individualized respiratory medicine.

## Figures and Tables

**Table 1:** Comparative Immunopathology of COPD, Asthma, and IPF. This table summarizes the major immunopathological differences and similarities between Chronic Obstructive Pulmonary Disease (COPD), Asthma, and Idiopathic Pulmonary Fibrosis (IPF), with respect to their inflammatory profiles, cellular mediators, biomarkers, and therapeutic implications.

Feature	COPD	Asthma	IPF
Primary trigger	Cigarette smoke, pollutants	Allergens, viral infections, irritants	Repetitive epithelial injury, aging, genetic factors
Main inflammatory cells	Neutrophils, macrophages, CD8+ T cells	Eosinophils, Th2 cells, mast cells	Epithelial cells, fibroblasts, alternatively activated macrophages
Key cytokines/mediators	TNF-α, IL-1β, IL-6, IL-8, CXCL1, MMP-9	IL-4, IL-5, IL-13, TSLP, IL-33	TGF-β1, PDGF, IL-13, IL-33, CCL18, alarmins (HMGB1)
Inflammation type	Type 1/neutrophilic	Type 2/eosinophilic (mostly)	Low-grade, profibrotic, dysregulated immune repair
Airway remodeling	Bronchiolar narrowing, emphysema	Subepithelial fibrosis, smooth muscle hypertrophy	Honeycombing, fibroblastic foci, loss of alveolar architecture
Biomarkers	CRP, fibrinogen, neutrophils, eosinophils (subset)	FeNO, eosinophils, periostin, IgE	KL-6, SP-A/D, MMP-7, CCL18
Steroid responsiveness	Low (except eosinophilic phenotype)	High in most cases	Minimal to none (in classic IPF)
Targeted therapies	LABA/LAMA, PDE4 inhibitors, anti-IL-5 (select cases)	ICS, anti-IL-5, anti-IL-4Rα, anti-IgE	Anti-fibrotics (nintedanib, pirfenidone), trials ongoing for anti-TGF-β
Role of adaptive immunity	CD8+ T cells, B cell follicles	Th2 cells, IgE-producing B cells	Th2, Th17 cells, Tregs, possible autoimmune elements
Senescence/Aging	Contributes to pathogenesis	Less prominent	Strongly implicated (telomere shortening, epigenetics)

**Table 2:** Biologic Therapies in Severe Asthma.

Biologic (Target)	Mechanism/Target Pathway	Patient Selection Biomarkers	Key Clinical Trials	Main Clinical Benefits
Omalizumab (anti-IgE)	Binds free IgE, prevents interaction with FcεRI on mast cells/basophils	Elevated total serum IgE (30–1500 IU/mL, depending on body weight); sensitization to perennial allergen	INNOVATE, EXALT; multiple real-world studies	↓ Exacerbations, ↓ ICS use, ↑ QoL, benefit in allergic asthma
Mepolizumab (anti–IL-5)	Neutralizes IL-5, reduces eosinophil survival/activation	Blood eos ≥ 150 cells/µL at screening or ≥300 cells/µL in previous year (though benefits extend beyond these thresholds)	DREAM, MENSA, SIRIUS	↓ Exacerbations, ↓ OCS use, ↑ FEV1
Reslizumab (anti–IL-5)	Neutralizes IL-5 (IV administration)	Blood eos ≥ 400 cells/µL (trial inclusion); benefits observed also at lower thresholds	BREATH, phase III studies	↓ Exacerbations, ↑ FEV1, improved asthma control
Benralizumab (anti–IL-5Rα)	Induces eosinophil and basophil depletion via ADCC	Blood eos ≥ 300 cells/µL; efficacy extends beyond this cut-off	SIROCCO, CALIMA, ZONDA	↓ Exacerbations, ↓ OCS dependence, ↑ FEV1
Dupilumab (anti–IL-4Rα)	Blocks IL-4 and IL-13 signaling (shared receptor)	Eosinophilic asthma, OCS-dependent asthma, uncontrolled asthma with/without atopy	LIBERTY ASTHMA QUEST, VENTURE	↓ Exacerbations, ↑ FEV1, ↓ OCS, effective in both allergic and non-allergic
Tezepelumab (anti-TSLP)	Blocks TSLP, upstream alarmin	Broad efficacy regardless of eosinophil count, FeNO, or IgE	PATHWAY, NAVIGATOR	↓ Exacerbations, ↑ FEV1, effective in T2-high and T2-low
Itepekimab (anti-IL-33)	Blocks IL-33 signaling, dampening type 2 response	Under clinical investigation; not yet approved	Phase II studies (e.g., NCT03469934)	Promising reduction in exacerbations; role not yet established

↑ indicates an increase; ↓ indicates a decrease.

**Table 3:** Biologic and Targeted Therapies Evaluated in COPD.

Therapy (Target)	Mechanism/Target Pathway	Key Clinical Trials	Main Results	Status
Dupilumab (anti–IL-4Rα)	Blocks IL-4 and IL-13 signaling	NOTUS	↓ Exacerbations (30–34%), ↑ lung function, ↑ QoL in eosinophilic COPD	FDA approved 2023
Benralizumab (anti–IL-5Rα)	Depletes eosinophils via ADCC	ABRA, GALATHEA, TERRANOVA	ABRA: ↓ treatment failure (OR 0.26). Phase III: primary endpoints not met, benefit in subgroups	Not approved
Mepolizumab (anti–IL-5)	Neutralizes IL-5, reduces eosinophil survival	METREX, METREO	METREX: modest ↓ exacerbations in eosinophilic COPD. METREO: no consistent benefit	Not approved
Ensifentrine (dual PDE3/4 inhibitor)	Bronchodilator + anti-inflammatory via PDE3/4 inhibition	ENHANCE-1, ENHANCE-2	↓ Exacerbations (~40%), ↑ FEV1	Phase III positive, under review
Itepekimab (anti–IL-33)	Blocks IL-33 signaling	Ongoing phase II/III trials	Promising reduction in exacerbations in early-phase studies	Investigational
Astegolimab (anti-ST2)	Blocks IL-33 receptor (ST2)	Early-phase trials	Preliminary efficacy, data limited	Investigational
IL-17A inhibitors	Block IL-17A signaling	Phase II studies	Preliminary results, potential role in neutrophilic COPD	Investigational

↑ indicates an increase; ↓ indicates a decrease.

**Table 4:** Emerging and Targeted Therapies in Idiopathic Pulmonary Fibrosis (IPF).

Therapy/Agent	Target/Mechanism of Action	Clinical Development	Key Findings
Nintedanib	Tyrosine kinase inhibitor (VEGFR, FGFR, PDGFR) → reduces fibroblast proliferation & ECM deposition	Approved (Phase III, INPULSIS trials)	Slows FVC decline; no reversal of fibrosis.
Pirfenidone	Anti-fibrotic & anti-inflammatory; suppresses TGF-β, reduces fibroblast activation & cytokines	Approved (Phase III, ASCEND, CAPACITY)	Slows FVC decline; improves progression-free survival.
Pamrevlumab	Anti-CTGF monoclonal antibody	Phase III (ZEPHYRUS-1: negative; ZEPHYRUS-2 stopped)	Failed to reduce FVC decline; highlights redundancy of fibrogenic pathways.
Ziritaxestat	Autotaxin inhibitor → ↓ LPA signaling and fibroblast activation	Phase III (ISABELA 1/2: discontinued)	Ineffective, safety concerns; program terminated.
Admilparant (BMS-986278)	LPA1 antagonist → blocks LPA-mediated fibroblast activation & fibrosis signaling	Phase III ongoing (IPF, PPF)	Promising biomarker modulation; early data suggest reduced disease progression.
Treprostinil (inhaled)	Prostacyclin analogue → vasodilation, anti-inflammatory, anti-fibrotic effects	Phase III (TETON trials ongoing)	Improved FVC in PPF; potential add-on to antifibrotics.
Integrin antagonists	Block αvβ6/αvβ1 integrins → reduce TGF-β activation and ECM remodeling	Phase II trials ongoing	Early efficacy signals; further validation required.
CAR-T (FAP-targeted, LNP-mRNA)	In vivo transient CAR-T therapy targeting fibroblast activation protein (FAP)	Preclinical (mouse models)	Ablates activated fibroblasts; restores alveolar architecture & ECM balance.
Rentosertib (ISM001-055)	TNIK inhibitor, AI-discovered → modulates Wnt/β-catenin and pro-fibrotic signaling pathways	Phase IIa clinical trial	Favorable safety; early signals of slowed FVC decline in IPF patients.

↓ indicates a decrease.

## Data Availability

Not applicable.
